# Anti-hLAMP2-antibodies and dual positivity for anti-GBM and MPO-ANCA in a patient with relapsing pulmonary-renal syndrome

**DOI:** 10.1186/1471-2369-12-26

**Published:** 2011-06-08

**Authors:** Christoph Etter, Ariana Gaspert, Stephan Regenass, Rudolf P Wüthrich, Thomas Kistler, Renate Kain, Clemens D Cohen

**Affiliations:** 1 Division of Nephrology, University Hospital Zurich, Zurich, Switzerland; 2 Institute of Physiology, University of Zurich, Zurich, Switzerland; 3 Department of Surgical Pathology, University Hospital Zurich, Zurich, Switzerland; 4 Division of Clinical Immunology, University Hospital Zurich, Zurich, Switzerland; 5 Division of Nephrology, Kantonsspital Winterthur, Winterthur, Switzerland; 6 Department of Pathology, Medical University of Vienna, Vienna, Austria

## Abstract

**Background:**

Pulmonary-renal syndrome associated with anti-glomerular basement membrane (GBM) antibodies, also known as Goodpasture's syndrome, is a rare but acute and life-threatening condition. One third of patients presenting as anti-GBM antibody positive pulmonary-renal syndrome or rapidly progressive glomerulonephritis are also tested positive for anti-neutrophil cytoplasmic antibodies (ANCA). Whilst anti-GBM disease is considered a non-relapsing condition, the long-term course of double-positive patients is less predictable.

**Case Presentation:**

We report a patient with such dual positivity, who presented with pulmonary hemorrhage, crescentic glomerulonephritis and membranous nephropathy. Plasmapheresis in combination with immunosuppresive therapy led to a rapid remission but the disease relapsed after two years. The serum of the patient was tested positive for antibodies to human lysosomal membrane protein 2 (hLAMP2), a novel autoantigen in patients with active small-vessel vasculitis (SVV). The anti-hLAMP2 antibody levels correlated positively with clinical disease activity in this patient.

**Conclusion:**

We hypothesize that this antibody may indicate a clinical course similar to ANCA-associated vasculitis in double-positive patients. However, this needs to be confirmed on comprehensive patient cohorts.

## Background

Anti-GBM disease, also known as Goodpasture's syndrome, is a paradigm for an autoimmune disease: The antigenic epitope in the non-collagenous region of the alpha 3 chain of type IV collagen [α3(IV)NC1] is well defined, and the confined expression of this collagen to glomerular and alveolar basement membranes leads to the organ specificity of the disease [1]. In renal biopsies linear positivity for immunoglobulin G (IgG) along the GBM indicates the direct pathogenetic relevance of the antibody. Interestingly, up to a third of patients with anti-GBM disease are also positive for ANCA, mainly with specificity to myeloperoxidase (MPO) [2-6]. This latter antibody is commonly associated with microscopic polyangiitis and to a lesser extent with granulomatosis with polyangiitis (Wegener's). Both are ANCA-positive SVV with frequent renal involvement as crescentic glomerulonephritis without prominent Ig deposition (pauci-immune CGN). The relatively high incidence of such dual positivity indicates a pathogenetic link, which still has to be unravelled. It is tempting to speculate on ANCA-associated mechanisms leading to the exposure of the otherwise hidden GBM-antigen [1,3]. Some reports on a sequential positivity of ANCA followed by anti-GBM antibodies support this hypothesis but other reports also describe the opposite sequence [7-9]. Controversies exist on the course of disease of double-positive patients. Older studies reported a favourable course [10] but more recent reports conclude that renal prognosis is comparable to anti-GBM disease [3,11]. Whilst anti-GBM disease is generally considered a non-relapsing illness, ANCA-positive SVV has a relevant risk of relapses demanding maintenance therapy after induction of remission [12]. In double-positive patients both relapsing and non-relapsing courses of disease can be observed [5,6]. Therefore, an indicator for relapsing disease in double-positive patients is awaited.

This report summarizes our experience on diagnosis and treatment of a patient with pulmonary-renal syndrome (PRS), relapsing CGN with subepithelial immune deposits and serological dual positivity for both anti-GBM antibodies and MPO-ANCA, who was also tested positive for novel SVV-associated antibodies against hLAMP2.

## Case presentation

**Figure 1:**
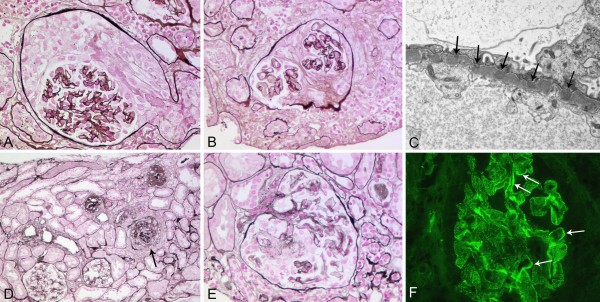
**Renal biopsy findings**. The first biopsy (corresponding to month 0 in table 1) showed cellular crescents (A) in two and fibrocellular crescents (B) in three out of nine glomeruli, consistent with crescentic glomerulonephritis with moderate activity and moderate chronicity (Silver methenamine stain; original magnification, (A) ×200, (B) ×180). The glomerular basement membrane revealed multiple small holes, no spikes. Immunofluorescence study results were negative but on electron microscopy multiple small subepithelial deposits (arrows) indicative of membranous nephropathy stage 1 were visible (C, original magnification, ×10.500). In the second biopsy (corresponding to month 23 in table 1) widespread global sclerosis was present with sclerosis of 15 of 32 glomeruli and crescents in 5 glomeruli: two segmental fibrous crescents, two fibrocellular (one gobal (arrow in D)), one segmental cellular crescent, with focal interstitial fibrosis and tubular atrophy, comprising 25% of the cortex, consistent with late ANCA-associated sclerosing glomerulonephritis with minimal activity and severe chronicity (Silver methenamine stain; original magnification, (D) ×50, (E) ×160). Immunofluorescence studies showed finely granular glomerular basement membrane positivity for IgG (2+) corresponding to the previously diagnosed membranous nephropathy and a segmental linear (arrows) GBM positivity (F original magnification, ×280).

A non-smoking, 52-year-old woman presented to her general practitioner with headache, fever and right-sided thoracic pain. The chest radiograph showed a pulmonary infiltrate of the right lower lobe. Antibiotic therapy was initiated. Due to persistent fever a chest computed tomography (CT) was performed two weeks later, which showed low grade but diffuse ground-glass infiltration beside previously described bronchiectasis. Alveolar hemorrhage was documented by bronchoscopy. The histologic examination of a lung biopsy showed alveolar siderophages and focal chronic lymphocytic infiltration; no immunofluorescence was performed. Consecutive decline of kidney function completed the clinical picture of a PRS and the patient was referred to a renal division. Laboratory values at the time of referral are given in Table 1. The serum tested positive for anti-GBM antibodies as well as ANCA by standard indirect immunofluorescence (ANA negative, anti-dsDNA 6 E/ml (normal < 20)). Subsequent tests revealed antibody reactivity against both, MPO and NC1 domain of type IV collagen. A renal biopsy was performed and documented a necrotizing extracapillary proliferative glomerulonephritis. There were nine glomeruli, one hyalinized, with five mostly segmental crescents (two cellular, three fibrocellular). A less well-preserved, frozen biopsy specimen did not show linear staining for human IgG by direct immunofluorescence. Similarly, all other immunoglobulins and complement factors were negative (IgA, IgM, Kappa, Lambda, C3, C1q). This was surprising since electron microscopy revealed small subepithelial deposits of a membranous nephropathy stage 1, without subendothelial or mesangial deposits (Figure 1, see legend for details). The co-occurance of CGN and membranous nephropathy is rare but has been described in detail previously [13,14].

**Table 1:** Laboratory results at initial presentation (month -1), admission to hospital (month 0, time of first renal biopsy), remission (month 1, 6, 12, 18), first relapse (month 23, time of second renal biopsy), and under therapy (month 25)

Time (months)	-1	0	1	6	12	18	23	25
**Renal Biopsy (no.)**		**1**					**2**	

Hemoglobin [11.7 - 15.3 g/dl]	9.4	**8.7**	9.2	11.7	12.6	14.1	**11**	12.9

C-reactive protein [< 5 mg/l]	84	**13**	2	< 3	< 3	< 3	**< 3**	< 3

Creatinine [44 - 80 μmol/l]	121	**428**	172	105	97	106	**165**	136

eGFR [> 60 ml/min]	41	**10**	27	48	53	48	**29**	35

Proteinuria [< 150 mg/d]	n.a.	**1700**	n.a.	130	110	139	**590**	230

MPO-ANCA [< 5 units/ml]	121	**58**	7	8	13	19	**53**	10

Anti-GBM [< 10 units/ml]	130	**68**	9	2	n.a.	n.a.	**23**	7

hLAMP-2 [units, see legend]	n.a.	**n.a**.	2	23	20	20	**31**	19

Normal values and units are given in squared bracket. eGFR = estimated glomerular filtration rate according to MDRD formula, n.a. = not available.

For detection of hLAMP-2 antibodies archival sera for different time-points were assayed. Briefly, the ELISA used human recombinant extracellular domain expressed in E. coli as substrate and the results were expressed as units derived from standard curve of a single strongly positive serum (negative < 22, possible 22 - 28, positive > 28)

**Figure 2:**
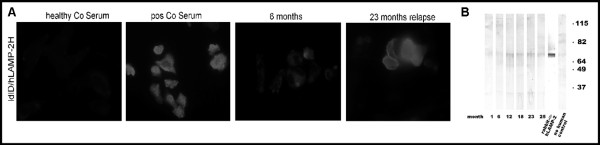
**Detection of anti-hLAMP2 antibodies**. A. The patient's serum at time of relapse (month 23) was tested positive by indirect immunfluorescence on ldlD cells stably expressing hLAMP-2 on the cell surface; whereas only minor staining was obtained during remission (month 6). Results of the serum from a healthy, negative control and a positive SVV-control are shown in picture 1 and 2, respectively. B. The serum was also tested positive by Western blot on E. coli expressed fusion protein. Lane 1-6: Serum of our patient at different time-points (one major band corresponding to the fusion protein at around 66 kD). Lane 7: rabbit anti-hLAMP-2 polyclonal serum (positive control). Lane 8: anti-human (negative control). All methods for detection of hLAMP-2 antibodies are published in detail elsewhere [15].

We concluded that the patient had double-positive Goodpasture's syndrome with falsely negative immunofluorescence due to a not perfectly preserved frozen biopsy specimen. Induction therapy was established with plasma exchange, pulse cyclophosphamide and prednisone [12]. Anti-GBM titers became negative, MPO-ANCA fell to 8 E/ml (normal < 5; Table 1). Cyclophosphamide was stopped after three months but we decided to pursue with maintenance therapy with azathioprine because of the aforementioned findings also consistent with SVV. However, on demand of the patient prednisone was rapidly tapered and azathioprine stopped after 15 months. Kidney function had recovered to an estimated glomerular filtration rate of 50 ml/min. Seven months after discontinuation of the maintenance therapy the patient presented with mild dyspnea and worsening kidney function as well as proteinuria and microhematuria with dysmorphic erythrocytes. Anti-GBM antibodies were found to be positive again (28 E/ml) and rising MPO-ANCA were detected (56 E/ml, Table 1). An initial chest CT showed regressive diffuse small pulmonary infiltration, but no signs of acute pulmonary hemorrhage. For the second time a kidney biopsy was performed and showed a mostly global sclerosing but also extracapillary proliferative glomerulonephritis again in combination with a membranous nephropathy (detailed in the legend of Figure 2). Focal interstitial fibrosis and tubular atrophy was also observed. A global finely granular and a discreet segmental linear IgG positivity was detected by immunofluorescence in the GBM (Figure 1).

As this patient presented with the rare combination of dual positivity for anti-GBM and MPO-ANCA with relapsing pauci-immune CGN we examined the serum at time of relapse for anti-hLAMP2 antibodies, a new class of antibodies associated with SVV [15]. The serum was indeed tested positive for anti-hLAMP2 antibodies. This prompted us to test also archival serum samples of this patient by enzyme-linked immunosorbent assay (ELISA), immunfluorescence on cells stably expressing hLAMP2 on the cell surface, and western blot (Table 1 and Figure 2). The serum concentration of anti-hLAMP2 antibodies correlated positively with disease activity (Table 1 and Figure 2). The lack of the typical intense global linear IgG staining of the glomeruli combined with rising antibody titers to MPO together with the presence of anti-hLAMP2 antibodies led us to judge the disease as relapsing ANCA-associated SVV; hence plasmapheresis was discontinued and cyclophosphamide with prednisone successfully reintroduced.

## Conclusion

So far, no conclusive maintenance therapy for patients with dual antibody specificity for NC1 and MPO has been established. This is at least in part due to the lack of understanding the sequential events leading to the development of both antibody types but, more importantly, due to failure to identify the disease defining antibody that will, ultimately, determine progression and outcome. Anti-hLAMP2 antibodies appear to indicate active SVV and may thus serve as an indicator for relapsing disease also in patients with dual positivity. This antibody reacts with an antigen expressed on endothelial cells and neutrophils that shares complete homology to the bacterial adhesin FimH [15]. It is reported to be present in 90% of cases with active phases of ANCA-associated necrotizing and crescentic glomerulonephritis and was also seen in a small proportion of patients with ANCA-positive Goodpasture's syndrome [15].

Several characteristics of this patient led us to test her serum for anti-hLAMP2 antibodies: First, Goodpasture's syndrome is considered a non-relapsing disease [5,6,11,16]. In this patient the disease relapsed after a clinical and serological remission. Second, two findings in the renal biopsies are also suggestive for a SVV-like disease: The initial biopsy did reveal different ages of glomerular lesions (cellular and fibrocellular crescents, detailed in the legend of Figure 1). This indicates a continuously evolving glomerulonephritis and not a sudden and defined initiation. Such diverse glomerular alterations are less common in acute anti-GBM disease [16]. The immunofluorescence of the initial kidney biopsy was not positive and the subsequent biopsy showed mostly a granular positivity for IgG corresponding to the membranous nephropathy and only a segmental and not the intense global linear positivity for IgG as in typical anti-GBM disease. In the literature most authors report strong linear positivity for IgG in double-positive patients [2,4,6], although a scanty positivity has also been described in a subset of patients [3]. The membranous nephropathy is another interesting aspect in our patient. Although glomerular immune deposits are found in about 50% of ANCA-associated CGN an isolated subepithelial localisation of the immune complex deposits is rarely observed. If present the combination of membranous nephropathy and CGN appears to be associated with high proteinuria and may accentuate the clinical course of CGN [13,14].

We concluded that the clinical course and the histology results are more suggestive for SVV than anti-GBM disease. This SVV-like course of disease can be seen in conjunction with the positivity for anti-hLAMP2 antibodies. One may hypothesize that the primary event is SVV with glomerular and alveolar damage and subsequent development of anti-GBM antibodies. However, this can only be tested on serial serum samples taken before disease manifestation and cannot be concluded from a case-report.

This case highlights the importance of testing for coexistence of different antibodies in patients with PRS, as it may be a common manifestation of both SVV and Goodpasture's disease [5,6,17]. However, since the best long-term treatment of double-positive patients is unknown, an indicator for a relapsing SVV-like course of disease would be helpful. Our case and its interpretation suggest that anti-hLAMP2 antibodies may indicate a SVV-like course of disease and therefore could be of clinical utility for the management of double-positive patients. However, this needs to be tested on adequate patient cohorts.

## Consent

Written informed consent was obtained from the patient for publication of this case report and any accompanying images. A copy of the written consent is available for review by the Editor-in-Chief of this journal.

## List of abbreviations

ANCA: anti-neutrophil cytoplasmic antibodies; CGN: crescentic glomerulonephritis; CT: computed tomography; GBM: glomerular basement membrane; hLAMP2: human lysosomal membrane protein 2; IgG: immunoglobulin G; MPO: myeloperoxidase; PRS: pulmonary-renal syndrome; SVV: small-vessel vasculitis

## Competing interests

The authors declare that they have no competing interests.

## Authors' contributions

AG interpreted the kidney biopsies and wrote the corresponding legend. SR analyzed the patient's serum for presence and specificity of ANCA and anti-GBM antibodies. TK first treated the patient and provided data about the history and laboratory results. RK analyzed the patient's serum for presence of anti-hLAMP2 antibodies and contributed to the manuscript. CE and CDC treated the patient, collected data and sera, and drafted the manuscript together with RPW. All authors read and approved the final manuscript.

## Pre-publication history

The pre-publication history for this paper can be accessed here:

http://www.biomedcentral.com/1471-2369/12/26/prepub
